# Book Reviews

**Published:** 1899-02

**Authors:** 


THE PRACTITIONER'S MANUAL. A condensed system of medical diagnosis and treatment. By CHARLES WARRENNE ALLEN, M. D., Consulting Genito-urinary Surgeon to the City (Charity) Hospital; Consulting Dermatologist to the Randall's Island Hospital; Attending Surgeon to the Good Samaritan Hospital, etc., etc. Octavo, pp. 85. New York: William Wood & Co. 1898.

This work presents to the medical profession a condensation of the practical features of text-books, manuals and monographs on medicine. It is alphabetically arranged, contains short dissertations upon most medical diseases, together with a great number of prescriptions specially collected from the most approved sources.

It affords the over-worked physician who must econimise time, an opportunity to get quick information on a subject that may be for the time being absorbing his thoughts. It begins with "abscess of the brain" and ends with "vertigo." A most complete index makes each and every subject readily accessible. It contains valuable hints as to diagnosis and treatment on almost every disease for which a physician may be consulted. It cannot take the place of text-books on medicine, nor of the several brochures thereof, neither does it aim to do so; but it affords a new way of utilising much that otherwise might escape observation and of getting at it quickly.

It cannot be denied that the market is overstocked with year-books, annuals and ready-reference volumes, all of which the profession is over solicited to purchase, but here is a unique practical book that cannot fail to prove of value to every purchaser.

MANUAL OF CHEMISTRY. A guide to lectures and laboratory work for beginners in chemistry. A text-book specially adapted for students of pharmacy and medicine. By W. SIMON, Ph. D., M. D., Professor of Chemistry and Toxicology, College of Physicians and Surgeons, Baltimore; Professor of Chemistry in the Maryland College of Pharmacy. New (sixth) edition. In one 8vo volume of 532 pages, with forty-six engravings and eight colored plates, illustrating sixty-four of the most important chemical tests. Philadelphia and New York: Lea Brothers & Co., Publishers. 1898.

The last edition of this excellent manual appeared in 1895. That a new one is demanded within three years is evidence of its usefulness and of the hold it has secured upon teachers, for this is a department of medical science that cannot expect to sell its literature to students, unless it is endorsed and recommended by teachers. This edition follows the same general plan as the fifth, but it has been here and there freshened by such additions as the progress of the science has made necessary. The needs of the student are especially conserved in this work, and this applies equally to the student of pharmacy as of medicine. We know of no other work that so distinctly appeals to the necessities of these two classes of students or so pleasantly teaches the underlying principles of this so nearly exact, though often occult or dull, branch of medical science.

---

A TREATISE ON THE SCIENCE AND PRACTICE OF MIDWIFERY. By W. S. PLAYFAIR, M. D., LL. D., F. R. C. P., Emeritus Professor of Obstetric Medicine in King's College, London; Examiner in Midwifery to the Universities of Cambridge and London. Seventh American from the ninth English edition. In one 8vo volume of 700 pages, with 207 engravings and seven full-page plates. Philadelphia and New York: Lea Brothers & Co., Publishers. 1898.

For more than twenty years Playfair's treatise on obstetrics has occupied a foremost position in the estimation of teachers and practitioners as well as students of midwifery. Sixteen editions have been put forth in this country and in England, which is evidence of its merit, and is a record that few medical books can boast and, as far as we remember, is unique in obstetrical literature.

This edition presents a complete revision of the text, many parts of which have been rewritten, thus bringing every department forward to the present period. New illustrations have also been added to keep pace with progress, and some obsolete cuts have been eliminated for like reasons.

Taken altogether, Playfair is one of the safest guides in the field of obstetrics and in its practical presentation of the subject commends itself alike to teachers, obstetricians and students.

---

TRANSACTIONS OF THE AMERICAN SURGICAL ASSOCIATION. Volume XVI. Edited by DE FOREST WILLARD, A. M., M. D., Ph. D., Recorder for the Association. Octavo, pp. xlii—318. Philadelphia: William J. Dornan, Printer. 1898.

This most excellent association again presents to the profession of medicine an interesting volume of transactions. A superb portrait of Dr. D. Hayes Agnew, is published as a frontispiece. A picture of the D. Hayes Agnew memorial wing of the Pennsylvania Hospital is also published in this book. This building was erected at a cost of $175,000 and contains five departments—children's orthopedic, general surgical, gynecological, opthalmological and otological—besides dispensaries, laboratories, *x*-ray and photographic studios, sterilisation and instrument rooms, nurses' quarters, etc., etc. It contains 150 beds, has three clinical amphitheatres, seating from 125 to 275 students, and is fitted with the best devices for teaching and performing aseptic surgery. The Pennsylvania, though one of the oldest hospitals in the country, is made equal to the most modern by this splendid endowment.

The papers in the volume are up to the usual standard of this association, although the book is not quite as large as some of its predecessors. It should be in the hands of every surgeon.

Index-Catalogue of the Library of the Surgeon-General's Office, United States Army. Authors and Subjects. Second Series. Vol. III. C. Czygan. Washington: Government Printing Office. 1898.

This volume of 1,100 pages, includes 11,112 author-titles, representing 4,873 volumes and 10,690 pamphlets. It also contains 10,636 subject-titles of separate books and pamphlets and 34,314 titles of articles in periodicals. There are catalogued in it 677 medical portraits, under the heading, "Collection of portraits." The library now contains 127,938 bound volumes and 216,839 pamphlets. Dr. James C. Merrill, major and surgeon, U. S. Army, is the librarian, under whose direction this volume was prepared.

Diseases of Women: A Manual of Gynecology. Designed especially for the use of students and general practitioners. By Francis H. Davenport, M. D., Assistant Professor of Gynecology in the Medical Department of Harvard University, Boston. New (third) revised and enlarged edition. In one handsome 12mo volume of 387 pages, with 155 illustrations. Philadelphia and New York: Lea Brothers & Co.

This manual having progressed to its third edition its status appears to be fixed, and it remains for us merely to state the changes that have been made in this edition. The author has deemed it wise to include in this one surgical as well as nonsurgical methods of treatment, hence the scope of the manual has been enlarged in that direction. The book, however, does not pretend to aspire to the needs of the specialist, but modestly claims its originally chosen place among students and practitioners of medicine. Some of the old illustrations have been omitted and a few new pictures have been added, thus making it conform more nearly to the demands of the present time. All things considered, we regard this book entitled to a place in the foremost rank of works of its class.

